# Oxidative stress is two‐sided in the treatment of acute myeloid leukemia

**DOI:** 10.1002/cam4.6806

**Published:** 2024-05-08

**Authors:** Chenyang Fan, Xiangdong Yang, Lixiang Yan, Zhexin Shi

**Affiliations:** ^1^ First Teaching Hospital of Tianjin University of Traditional Chinese Medicine Tianjin China; ^2^ National Clinical Research Center for Chinese Medicine Acupuncture and Moxibustion Tianjin China

**Keywords:** acute myeloid leukemia, autophagy, chemoresistance, niche, oxidative stress, ROS

## Abstract

**Introduction:**

Oxidative stress caused by elevated ROS, as a novel therapeutic mechanism, has been implicated in various tumors including AML. AML cells are chronically under oxidative stress, yet overreliance on ROS production makes tumor cells increasingly vulnerable to further damage. Reducing the cytotoxic effect of ROS on normal cells while killing leukemia stem cell (LSC) with high levels of reactive oxygen species is a new challenge for oxidative stress therapy in leukemia.

**Methods:**

By searching literature databases, we summarized recent relevant studies. The relationship of ROS on AML genes, signaling pathways, and transcription factors, and the correlation of ROS with AML bone marrow microenvironment and autophagy were summarized. In addition, we summarize the current status of research on ROS and AML therapeutics. Finally, we discuss the research progress on redox resistance in AML.

**Results:**

This review discusses the evidence showing the link between redox reactions and the progression of AML and compiles the latest research findings that will facilitate future biological studies of redox effects associated with AML treatment.

**Conclusion:**

We believe that exploiting this unique oxidative stress property of AML cells may provide a new way to prevent relapse and drug resistance.

## 1 INTRODUCTION

Acute myeloid leukemia (AML) is a hematological malignancy associated with malignant clones of hematopoietic cells. Current treatments include chemotherapy, allo‐HSCT, and immunotherapy. However, treatment outcomes are poor, with a 5‐year survival rate of only 29.5%.^1^ Notably, relapse and drug resistance are the biggest challenges facing AML therapy, with relapse rates of leukemia (including allo‐HSCT) of up to 40%–50% in all age groups.^2^, ^3^ New drug venetoclax shows high remission rates, but has a short‐lived effect, is resistant, and eventually leads to refractory relapses.^4^ Therefore, more research and exploration are still needed to overcome the above problems.

Reactive oxygen species (ROS) are molecules with high oxidative activity.^5^ ROS include oxygen free radicals (O_2_^·−^, OH) and nonradical oxidants (H_2_O_2_ and ^1^O_2_).^6^ Mitochondria and nicotinamide adenine dinucleotide phosphate oxidases (NOX) are the two primary sources of endogenous ROS in tumor cells.^7^ The role of ROS in cells is paradoxical.^8^ ROS are critical in maintaining cell differentiation growth and homeostasis in vivo.^9^ Antioxidants such as GPx, SOD, catalase, and DNA repair proteins are the main force in cells to resist oxidative stress.^10^ An imbalance between ROS and antioxidants can induce oxidative stress, leading to oxidative damage to DNA, lipids, and proteins, thereby promoting the growth of tumor cells.^11^, ^12^ Paradoxically, pro‐oxidative stress has also been effective in selectively eliminating tumor cells.^13^, ^14^ Tumor cells, unlike normal cells, require higher levels of ROS for growth; however, their antioxidant capacity requires efforts to remove excess ROS while maintaining pro‐tumor signaling and antitumor cell apoptosis.^15^ Therefore, once ROS overproduction exceeds a certain threshold or the antioxidant system is damaged, it will lead to apoptosis of tumor cells.^14^ As AML primitive cells grow, ROS levels within the leukemia stem cell (LSC) increase significantly due to the collapse of antioxidant response factors.^16^ The two primary sources of ROS in tumor cells are the mitochondrial respiratory chain and NOX.^17^, ^18^ The NOX protein family consists of seven enzyme complexes, including NOX1‐5 and two related dual oxidases (DUOX1‐2).^19^ We review recent studies on oxidative stress and redox homeostasis in AML, with the urgency to find new therapies to target relapsed/chemoresistant AML cells susceptible to the latest drugs.

**FIGURE 1:**
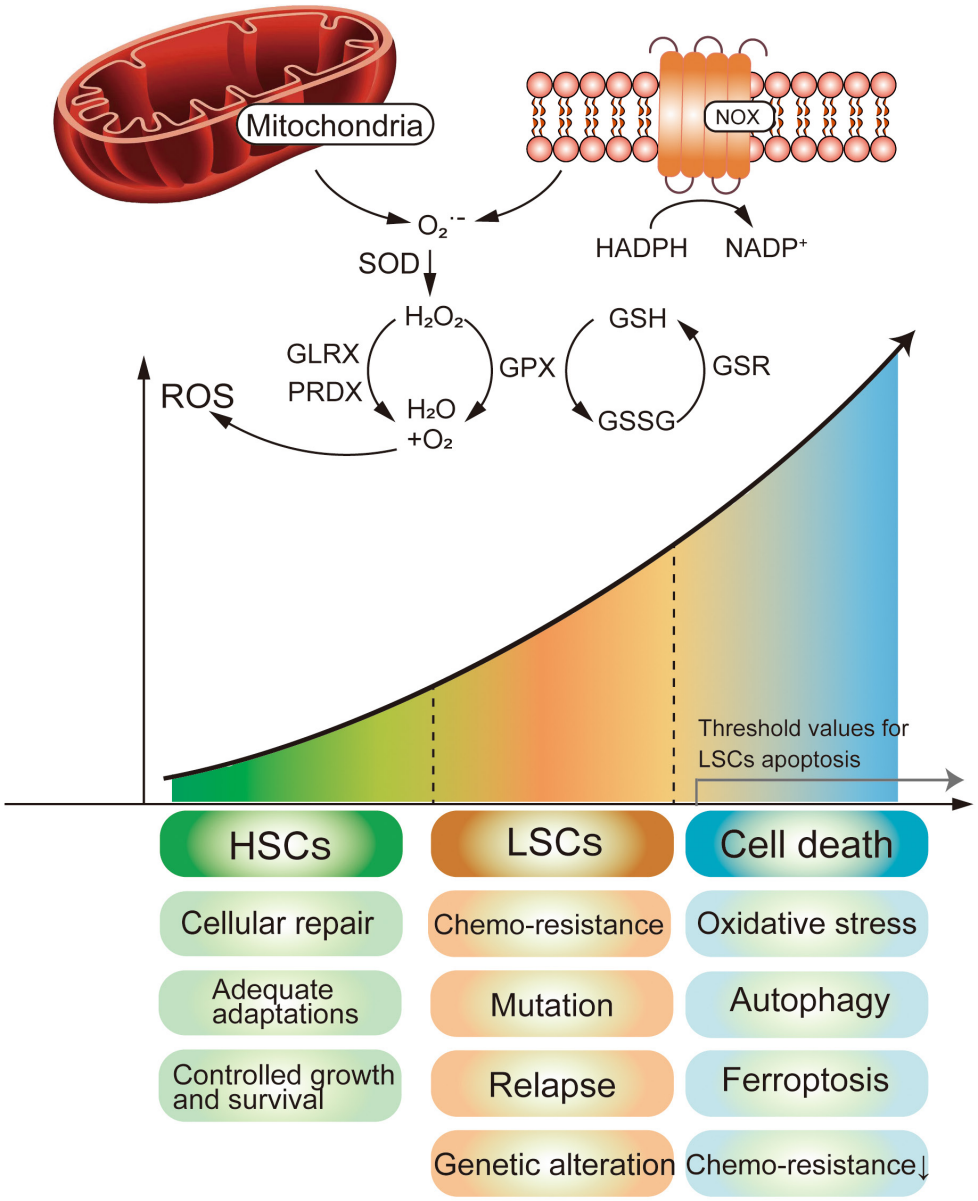
Overview of the intervention of ROS production and level changes on AML cell status figure. Mitochondria and NOX are the two primary sources of endogenous ROS in tumor cells. The level of ROS within LSCs is increased by the collapse of antioxidant response factors (GLRX, GPx, PRDX, and GSR). The conversion of GSH to GSSG can also regulate the level of ROS. When ROS in LSCs reach the apoptotic threshold, LSCs exhibit effects such as autophagy, iron toxicity, and drug sensitivity, which facilitate disease treatment. ^1^O_2_, singlet oxygen; GLRX, Glutaredoxin‐1; GPx, glutathione peroxidase; GSH, glutathione; GSR, glutathione reductase; GSSG, glutathione disulfide; H_2_O_2_, hydrogen peroxide; HSCs, hematopoietic stem cells; NADP+, nicotinamide adenine dinucleotide phosphate+; NADPH, nicotinamide adenine dinucleotide phosphate; O_2_^·−^, superoxide anion radical; PRDX, peroxiredoxins.

In the following figure, we can see the mechanism of ROS production with the regulation of oxidants and antioxidants. Also, we can understand the effect of changes in ROS levels on AML cells at different stages (Figure 1).

The cause of AML has not been elucidated and may be related to physical, chemical, biological, and genetic factors. We have found a significant association between oxidative stress and the pathogenesis of AML along with its treatment. Oxidative stress has now been shown to have a definite relationship with the process of MDS secondary to AML.^16^ We should try to inhibit AML progression by modulating the level of ROS. Early AML can be an antitumor by inhibiting ROS, and mid‐ to late‐stage AML can target oxidative stress to eliminate LSC.^20^ This review aims to discuss the dual nature of oxidative stress in AML, divided into four main aspects: (1) ROS‐mediated genetic alterations; (2) we summarize signaling pathways and transcription factors associated with oxidative stress and AML; (3) oxidative stress modulators in AML; and (4) the relationship of ROS with niches, autophagy, and AML resistance.

## 2 ROS‐MEDIATED AML GENE ALTERATIONS

### 2.1 Oxidation of DNA by ROS

ROS‐induced DNA damage and genome destabilization play a crucial role in the pathogenesis and development of various tumors, including AML.^21^ ROS induces DNA deoxyribonucleic acid single‐strand breaks (SSBs) and double‐strand breaks (DSBs). In both telomerase and alternatively lengthened telomere (ALT)‐positive carcinoma cells, ROS‐induced DNA injury is repairable.^22^ Breakage of DNA replication is a novel pathway to protect telomeres from ROS damage.^23^ ROS‐induced SSBs are the primary inducers of the R‐loop. SSBs can enhance DSB formation and facilitate DSB recovery by inducing the R‐loop. This discovery reveals an interplay between ROS‐induced outcomes.^23^ ROS induces efficient repair of telomeric DSBs. The R‐loop concentrates cockayne syndrome protein B (CSB) and RAD52 on ROS‐damaged telomeres.^24^ The CSB‐RAD52‐POLD3 axis mediates the telomeric breakage of DNA replication pathway that is triggered by ROS through the telomeric R‐loop. This axis is preferentially used for telomeric DNA repair in ALT‐positive tumor cells.^23^ Samassekou et al. investigated the presence of extrachromosomal telomeric repeat (ECTR) sequences in primary chronic myeloid leukemia (CML) cells and showed that ALT‐mediated telomere regulation is more prevalent than expected in CML.^24^ Alternative extension of telomere‐associated promyelocytic leukemia nucleosomes (APBs), a symbol of telomere maintenance, is associated with ALT.^25^ The ALT pathway is an appealing therapeutic target for the treatment of cancer. Activation of ALT has been demonstrated in CML, promyelocytic leukemia, and a variety of solid tumors, however, studies on ALT and AML have not been clarified. However, once we confirm the presence of ALT in AML cells, we can further develop inhibitors that target ALT. Thereby, ALT deficiency enhances DNA damage in LSC by high levels of ROS. The ultimate goal is to treat AML.

The preleukemic fusion genes (PFG) is a product of DNA damage and chromosomal translocations in HSC/hematopoietic progenitor cell (HSPC).^26^ PFG is one of the most significant clinical tests for leukemia diagnosis and treatment. The most common PFGs include BCR‐ABL and TEL‐AML1.^27^ The ROS‐induced DNA double‐strand break predicts that it has the potential to trigger PFGs.^28^ According to studies, ROS‐triggered PFG expression may also interfere with DNA repair.^21^, ^29^ Somsedikova et al. measured DSB levels with γH2AX/53BP1 in acute lymphocytic leukemia cells carrying BCR‐ABL or ETV6‐RUNX fusion genes and in cells not carrying PFG. The researchers found significantly higher levels of DSBs in cells bearing PFG.^30^ Furthermore, Sallmyr et al. found that FLT3‐ITD gene mutations induce a cycle of genomic instability that increases ROS leading to DSB and repair errors.^31^ Therefore, patients with PFG and genetic mutations in AML tend to have a poor prognosis.

### 2.2 Oxidation of lipids by ROS

ROS‐mediated oxidation of polyunsaturated fatty acids (PUFA) causes lipid peroxidation, the main products of which are malondialdehyde (MDA) and 4‐hydroxy‐2‐nonenal (HNE).^32^ According to studies, MDA and HNE were higher in the leukemia cohort compared with the normal cohort.^33^, ^34^ Research has shown that HNE reduces oxidative damage in tumor by reducing ROS production through activation of uncoupling Protein (UCP).^35^ In addition, excessive formation of HNE can affect mitochondrial function and eventually lead to cell death.^36^ On the contrary, HNE can reduce intracellular iron by activating the nuclear factor erythroid 2‐related factor 2 (Nrf2)/HO‐1 pathway. Thus, reducing the concentration of HNE favors the antitumor effect of ferroptosis.^37^ Another essential lipid peroxidation product, MDA, causes DNA damage by interacting with DNA.^38^

The lipid peroxidation (LPO) reaction mediated by ROS is one of the necessary links for the emergence of ferroptosis. Ferroptosis is a unique form of regulated cell death. Unlike other types of cell death, it is associated with multiple metabolic pathways, including iron, ROS, and lipid metabolism.^39^ Early AML cell death is induced by iron concentration.^40^ ROS‐induced oxidative stress exacerbates the production of lipid peroxidation. Studies have shown that NOX4 impairs mitochondrial metabolic functions through oxidative stress‐induced lipid peroxidation, thus promoting cellular ferroptosis.^41^ Early ROS can increase the intracellular iron content by inducing ferritin autophagy, which degrades iron‐storing macromolecular ferritin, promoting ROS accumulation and eventually cell death.^42^ Ferroptosis is a form of LSC self‐clearance. Ferroptosis combined with immunology and autophagy to inhibit AML drug resistance is now increasingly studied. Research on traditional Chinese medicine targeting ferroptosis to prevent AML relapse is also underway.

### 2.3 Oxidation of proteins by ROS

A variety of intracellular proteins contain thiol residues. The redox action of these thiols regulates the activity of a wide range of proteins associated with transcription, translation, and biological functions.^43^ Several proteins (activator protein 1, NF‐κB, protein kinase C, caspases, thioredoxin, and tumor protein 53) are regulated by thiol oxidation^44^ and play an essential role in AML.^45^, ^46^, ^47^ For example, glutathione (GSH) is a small molecule of thiol. GSH reacts directly or enzymatically with oxidizing substances. This reaction results in the oxidation of GSH to GSSG.^48^ Thus, GSH can directly quench free radicals and ROS. Nrf2 is oxidized via the thiol of Kelch‐like ECH‐associated protein 1 (Keap1).^16^ In response to oxidative stress, the Keap1‐Nrf2 complex segregates. Nrf2 migrates into the nucleus of anti‐AML cells, leading to metabolic reprogramming and immunodeficiency in this cell.^49^ Nguyen et al. demonstrated that Nrf2 could be inhibited by Venetoclax, a selective inhibitor of Bcl‐2, thereby preventing ROS from being neutralized,^50^ as an additional mechanism of AML activity inhibition by the combination of drugs. However, the adequate application of protein sulfation to AML is unclear and more studies are needed to explore the mechanisms.

Protein phosphatase 2A (PP2A) is a tumor suppressor that regulates and catalyzes subunit composition. It can inactivate several components of the signaling pathways required for tumor growth and survival, such as the ROS‐related protein kinase B (Akt), mitogen‐activated protein kinases (MAPK), and Wnt signaling pathways.^51^ PP2A blunting is frequently observed in multiple solid and non‐solid tumors (including AML), leading to sustained activation of survival pathways or inhibition of apoptosis.^52^ PP2A is currently considered a tumor suppressor in AML.^53^, ^54^ Therefore, targeting ROS to regulate the Akt/MAPK/Wnt signaling pathway can activate PP2A to inhibit AML cell survival, which is an effective strategy to treat AML.

## 3 EFFECTS OF OXIDATIVE STRESS‐INDUCED ALTERATIONS IN SIGNALING PATHWAYS AND TRANSCRIPTION FACTORS ON AML

### 3.1 Signaling pathways

The phosphatidylinositol‐3‐kinase/Akt/ mechanistic target of rapamycin (PI3K/Akt/mTOR) is the core pathway of hematopoietic cells. This pathway has critical functions in regulating proliferation, differentiation, and survival^55^. Activation of this pathway is present in 60% of AML patients. This activation appears to be associated with reduced overall survival.^56^ The PI3K/Akt/mTOR pathway is upregulated in LSCs, and Akt may influence oxidative phosphorylation (OXPHOS).^57^ The PI3K pathway plays an essential role in regulating the autophagic response of cells to changes in ROS levels. Its downstream Akt protein responds to increased ROS levels by activating the mechanistic target of rapamycin complex (mTORC) 1 and inhibiting autophagic gene expression.^58^ The adenosine monophosphate‐activated protein kinase (AMPK) counteracts the effect of Akt. MTORC1 and mTORC2 inhibit autophagy at moderate ROS levels, but at high ROS levels, mTORC2 can promote cellular senescence through autophagy.^59^ The rat sarcoma virus/rapidly accelerated fibrosarcoma/mitogen‐activated protein kinase kinase/extracellular signal‐regulated kinase (Ras/Raf/MEK/ERK) and PI3K/Akt/mTOR signaling pathways act in strong synergy to regulate tumor cell function.^60^ The downstream targets of both the access pathways involve forkhead box O (FOXO), cellular myelocytomatosis oncogene (c‐Myc) transcription factor, Bcl‐2, glycogen synthase‐activating enzyme 3(GSK3), and phosphofructokinase‐2(PFK2).^61^ SYK is a common target of both pathways. The expression of SYK was positively correlated with the activity of both pathways.^62^ This finding has particular implications for patients with FLT3 mutations and AML with high SYK activation. Because in the phosphorylated state, FLT3 binds to SYK through its C‐terminal SH2 structural domain, thereby increasing FLT3 activity. This cooperative activation of FLT3 and SYK leads to the expression of c‐Myc.^63^ Therefore, more in‐depth studies can be conducted on SYK targeting the treatment of AML with FLT3 mutation.

**FIGURE 2:**
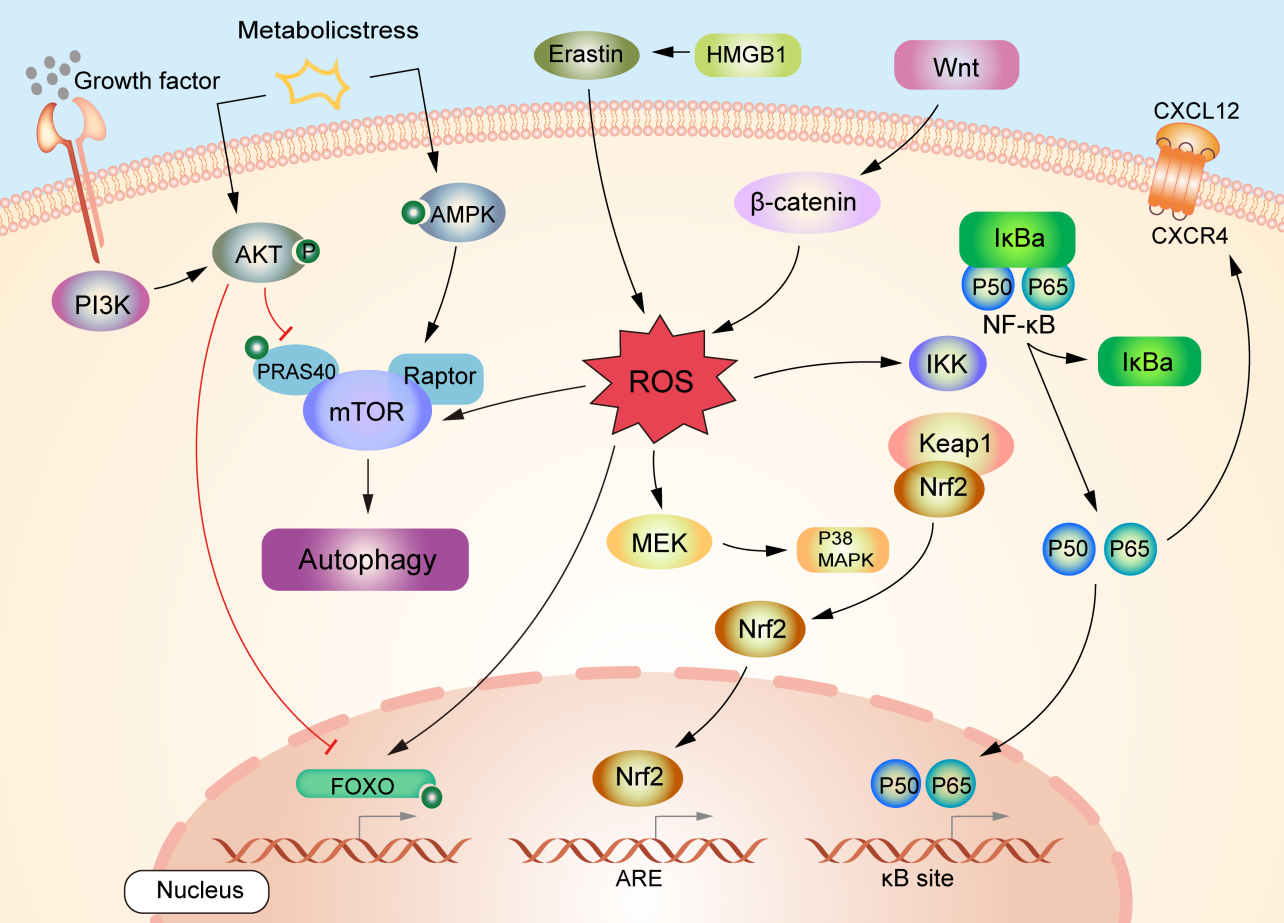
Schematic diagram of the main signaling pathways associated with oxidative stress and AML. Excessive production of ROS leads to an imbalance in oxidation/antioxidant production, which causes various signaling pathways to regulate the process of oxidative stress. Activating Keap1/Nrf2/ARE and PI3K/Akt/FOXO signaling pathways induces antioxidant expression and brings about antioxidative stress properties. Additionally, Wnt/β‐catenin, HMGB1, and NF‐κB signaling pathways are also involved in the formation of oxidative stress, and inhibition of these signaling pathways may exercise a protective effect against oxidative stress. Inhibition of NF‐κB transcription through oxidative stress inhibits CXCR4 expression and prevents AML recurrence to some extent. The PI3K pathway is necessarily important in regulating the autophagic response of cells to changes in the ROS levels. mTOR can promote cellular senescence through autophagy at high ROS levels. ARE, antioxidant response element; CXCL12, C‐X‐C motif ligand 12; CXCR4, C‐X‐C chemokine receptor 4; HMGB1, high‐mobility group box 1; IKK, ikappaB kinase; IκBα, ikappaB‐alpha; mTOR1, mechanistic target of rapamycin 1; PRAS40, 40‐kDa proline‐rich Akt substrate.

Wnt/β‐catenin signaling pathway is essential for hematopoietic stem cell expansion.^64^ The Wnt/β‐catenin signaling pathway is upregulated in inflammation, oxidative stress, and multiple cancers. Zhang et al. showed that Wnt/β‐catenin signaling induces HSC oxidation by promoting intracellular ROS production.^64^ It can be hypothesized that excessive ROS levels within the LSC are also generated through the Wnt/β‐catenin pathway, thus promoting AML progression. On the contrary, in AML, Wnt/β‐catenin inhibitors reversed Murine double minute X (MDMX)‐induced early LSC progression. MDMX is overexpressed in the majority of LSCs. MDMX overexpression increases the number of pre‐AML stem cells and disease progression.^65^ Therefore, Wnt/β‐catenin inhibitors for AML have research value. Dysregulation of the JAK/STAT pathway has been demonstrated in many hematological malignancies. It has been identified as a potent target for AML chemoresistance.^66^ Studies have shown that the JAK/STAT pathway can be activated by interferons and ROS.^67^ Therefore, whether oxidative stress can be used to overcome AML treatment resistance by modulating the JAK/STAT pathway needs future research. The following figure provides a schematic overview of the signaling pathways associated with ROS production and inhibition in AML (Figure 2).

### 3.2 Transcription factors

In addition to signaling pathways, some transcription factors are also sensitive to ROS, and transcriptional modifications of these related transcription factors can affect AML regression. FOXO is an important transcription factor. FOXOs have been identified as mediators of HSC resistance to oxidative stress.^68^ Meanwhile, high levels of ROS ablated the FOXO isoforms.^69^ Because Akt ubiquitinates FOXO and induces FOXO reverse transport. Long et al. showed that tyrosine kinase inhibitors (TKIs) resulted in the upregulation of histone deacetylase 8 (HDAC8) levels. This reaction is mediated by FOXO1 and FOXO3. Upregulated HDAC8 deacetylates and inactivates tumor protein 53, leading to TKI resistance to AML treatment.^70^ Therefore, it is reasonable to hypothesize that toxic amounts of ROS would increase AML cell drug sensitivity by inhibiting FOXO transcription. NF‐κB and ROS levels in AML cells have antagonistic effects on each other. ROS can inhibit NF‐κB synthesis by regulating IκBα, Ubc12, and IKK.^71^ Meanwhile, NF‐κB‐related pathways can also affect ROS levels by increasing the expression of antioxidant proteins.^71^ Pallarès et al. showed that LSC hijacks the adhesion mechanism of HSCs/HSPCs by upregulating the expression of CXCR4, vla4, and cd44,^72^ thus causing damage to normal bone marrow hematopoiesis. Excessive NF‐κB transcription within LSC significantly upregulated the expression of cellular CXCR4 gene by 2–3 fold.^73^ Inhibition of NF‐κB transcription through oxidative stress inhibits LSC adhesion and prevents AML recurrence to some extent.^74^ In addition to this, modulation of metabolic competition can also cause ROS within the LSC to reach the toxicity threshold, indirectly leading to LSC apoptosis.^75^ Erdem et al. suggested that Pyruvate dehydrogenase kinases (Pdks) are essential factors in the genesis of glycolysis.^76^ AML cells derive more energy to maintain their activity from ATP produced by OXPHOS and less from glycolysis. Blocking glycolysis can bring the ROS content in LSC to the toxicity threshold. Consequently, inhibition of PDk1 can attenuate the proliferative capacity of leukemia cells. This is associated with a decrease in BCL2 and BCL‐XL expression, increased PARP and Caspase‐mediated apoptosis, and a loss of autophagy regulators.^76^

As we have concluded above, high ROS cause Nrf2 to enter the nucleus of antitumor cells, leading to their metabolic reprogramming and immunodeficiency. At the same time, Nrf2 is a major regulator against oxidative stress.^77^ Nrf2 translocates to the nucleus and binds to the antioxidant response element (ARE) sequence. Both are reduced to cysteine for GSH synthesis.^78^ Thus, Nrf2 agonists effectively inhibit AML cell proliferation, activation, and resistance to oxidative stress. The high‐mobility group box 1 (HMGB1) transcription factor is involved in chromatin remodeling, DNA recombination, and repair processes.^79^ HMGB1 is produced in the cytoplasm, is readily expressed on the cell surface membrane, or diffuses into the extracellular space via multiple cellular stressors. Erastin is a ferroptosis activator. Its induced ferroptosis enhanced the sensitivity of AML cells (HL‐60/NRASQ61L) to chemotherapy.^80^ HMGB1 knockdown reduces Erastin‐promoted iron‐mediated ROS production and cell death. HMGB1 is a novel regulator of iron toxicity via the RAS‐JNK/p38 pathway and is a potential drug target for leukemia therapeutic intervention.^81^ Heat shock transcription factor 1 (HSF1) plays an essential role in the regulation of HSC protein homeostasis. However, when HSF1 is present in AML cells, HSF1 adapts tumor cells to the imbalance of DNA, and protein and energy‐consuming metabolic signals, a phenomenon known as “non‐oncogenic gene addiction.” Moreover, HSF1 enhances OXPHOS activity in LSC, which is favorable for LSC proliferation. Thus, HSF1 is a promising target for the treatment of AML. Dong et al. found that inhibition of HSF1 did not affect the protein homeostasis of HSC, which may be related to the redundancy of the mechanism of action of the HSF family on HSC.^82^ ZDHHC21 palmitoyltransferase is a critical regulator of OXPHOS hyperactivity in AML cells. Interestingly, FLT3‐ITD+ AML cells expressed significantly higher levels of ZDHHC21 and exhibited better sensitivity to ZDHHC21 inhibition.^83^ Inhibition of OXPHOS by targeting ZDHHC21 significantly eradicated LSC in relapsed/refractory (r/r) AML and enhanced the efficacy of chemotherapy. In conclusion, this finding reveals a novel biological function of ZDHHC21 in regulating AML OXPHOS. However, a blanket reduction in OXPHOS levels within the LSC may not lead to an excellent prognosis for the patient. This is because of the heterogeneity within LSC. Some of these LSCs proliferate positively correlated with the degree of OXPHOS, while others are in a state of OXPHOS (low) miR‐126(high) and show forced stemness and quiescent features. miR‐126(high) LSCs are enriched at diagnosis and relapse of chemotherapy‐refractory AML. Consequently, prognosis should still be determined by evaluating other metrics (e.g., MiR‐126) at low OXPHOS.^84^

## 4 OXIDATIVE STRESS AND BONE MARROW NICHE

**FIGURE 3:**
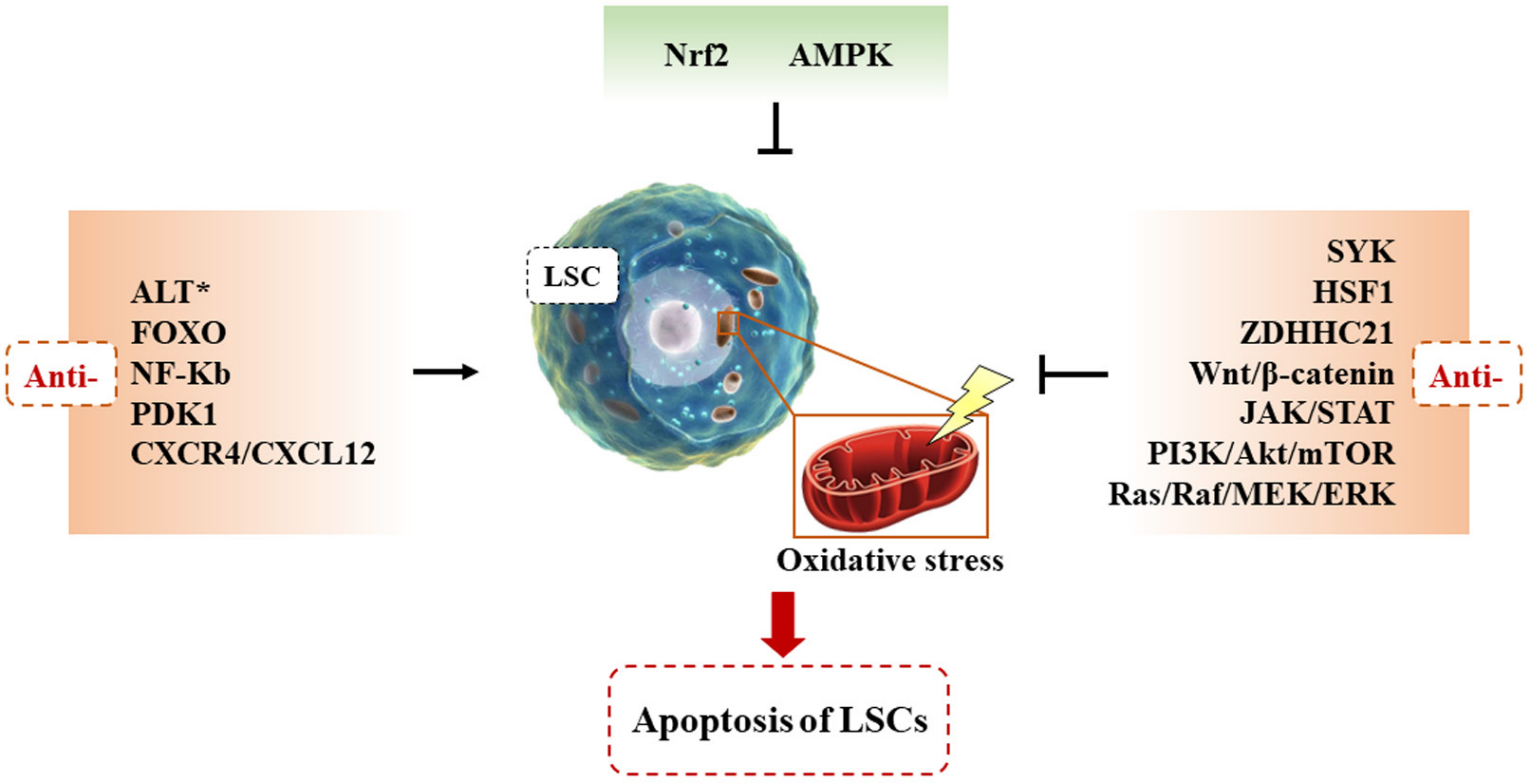
Schematic representation of critical molecules and pathways associated with LSC and their interactions with oxidative stress. Promoting Nrf2 or AMPK activity inhibits LSC oxidative stress, leading to apoptosis of LSCs. Inhibition of ALT, FOXO, NF‐Κb, PDK1, or CXCR4/CXCL12 activities served to promote LSC oxidative stress, leading to apoptosis of LSCs. By inhibiting SYK, HSF1, or ZDHHC21 molecular activities and signaling by Wnt/β‐catenin, JAK/STAT, PI3K/Akt/mTOR, or Ras/Raf/MEK/ERK pathways, oxidative stress can be suppressed, leading to apoptosis of LSCs. * Representing the role of this molecule or pathway on the oxidative stress response in LSC has not been demonstrated.

In the normal bone marrow (BM) niche, high levels of ROS are detrimental to HSPC in the niche and require the addition of antioxidants to rescue them.^85^ And the oxygen content in the leukemic niche promotes the synthesis of HIF‐1a, which further induces the upregulation of CXCR4 on the surface of LSCs. Through the interaction between CXCL12 and CXCR4, the adhesion force of LSCs is enhanced, allowing LSCs to anchor to the BM niche and remain quiescent, resulting in AML that is highly susceptible to relapse.^86^ Recent studies have shown that HSC obtains energy mainly through anaerobic enzymes, while LSC maintains survival mainly through mitochondrial oxidative respiration.^87^ In AML LSCs, mitochondria from bone marrow mesenchymal stem cells (BMSCs) can be transferred into LSCs via AML‐derived tunneling nanotubes. This process is dependent on NOX‐dependent oxidative stress‐mediated ROS production.^87^ Therefore, more energy is provided to the LSC via mitochondrial OXPHOS. However, this phenomenon was not observed in HSCs. Thus, blockage of the mitochondrial respiratory pathway of LSCs may contribute to the inhibition of LSCs themselves. Tang et al. showed that cytokines (SKP1, CUL1, F‐box protein, CXCL12, and IL‐7) were reduced in the niche due to chemotherapy‐induced BM niche by increasing intracellular ROS levels and inducing apoptosis in HSCs. This process disrupts the reconstruction of the hematopoietic system.^88^ However, the high levels of ROS caused by pro‐oxidant chemotherapy not only directly damage the HSCs, but also disrupt the BM niche where the HSCs are located.^89^ Then how exactly to reduce the cytotoxic effect of ROS on HSC while killing LSC with high levels of ROS is a challenge for the oxidative stress treatment of leukemia. Finally, the authors summarize the critical molecules and pathways associated with LSC and how they interact with oxidative stress (Figure 3).

## 5 ROS AND AUTOPHAGY IN THE TREATMENT OF AML

It has been recently shown that autophagic responses can be induced through oxidative stress. Conversely, ROS can also be reduced by autophagy. Autophagy can remove oxidatively damaged proteins and also ROS‐producing organelles (e.g., mitochondria and peroxisomes) to limit further ROS production.^90^ Autophagy acts critically in protecting HSC from oxidative damage and maintaining its stem cell characteristics.^91^ However, autophagy also has two sides, over‐autophagy can contribute to cell death.^92^ It is common knowledge that ROS is an essential factor in the induction of autophagy.^73^ An increase in ROS can induce autophagy through multiple signaling pathways, such as PI3K/Akt, AMPK, JNK, ERK, ATG4, and others.^93^ The level of autophagy in LSCs that depend on mitochondrial respiration may theoretically be higher than in HSCs that rely on glycolysis.^94^, ^95^ However, the hypothesis still needs to be confirmed by further research. Notably, it is essential that we grasp the precise level of ROS in order to induce autophagy in LSCs only. Currently, elimination of LSCs by autophagy without affecting normal hematopoietic cells has become a hot research topic. Combined with the above studies, it is also feasible to intervene ROS as a pathway to achieve this goal.

## 6 TARGETING REDOX ALTERATIONS IN AML

The induction of ROS is an essential mechanism factor for the treatment of AML drugs.^96^ Researchers have conducted numerous studies using ROS‐inducer‐mediated mechanisms to modulate oxidative stress responses in LSCs, thereby inducing apoptosis, necrosis, and autophagy. The authors summarized the mechanisms of ROS‐related treatable AML drugs (Table 1).

**TABLE 1:** ROS‐inducing agents used in AML‐derived cell lines.

Compounds	Cell lines	Major outcomes	Mechanisms	References
DNT	OCI‐AML2, OCI‐AML3, KG1A	↑NKG2D, DNAM‐1	Oxidative stress STING/cGAS activate	[99]
Actinomycin D	U994	↑ TP53, p21, Serpine‐1, PML NBs ↓ E2F	ROS/PML/TP53 activate Oxidative stress	[106]
Lestaurtinib	MOLM‐14, MV‐4‐11	↓ γH2AX	ROS/STAT5/RAC1/NADPH apoptosis	[31]
Brusatol	KG‐1, THP‐1, MOLM 13, MOLM13‐TKIR, MV411	↓ HO‐1, GPx	ROS/HO‐1/NrF2 apoptosis	[115]
4‐HPR	OCI‐AML2	↓ NF‐κB	Oxidative stress	]^116^]
Penfluridol	HL‐60, U937, MV4‐11	↑caspase‐3, PP2A, AVO ↓p62	ROS‐mediated autophagy PP2A/MAPK activate	[57]
Melatonin	ML‐1, HL‐60, MOLM‐13, MV4‐11	↑PARP ↓GSH, GSSG	Oxidative stress	[117]
APR‐246	HL60, MOLM14, SET2, MV4‐11, OCI‐AML2, OCI‐AML3, K562, THP1, UT7‐EPO, SKM1, NB4, KASUMI AML	↑lipid peroxides ↓GSH	Oxidative stress ferroptosis	[122]
TH1579	HL60, KASUMI, KG1A, MV4‐11, THP‐1, KBM‐3, PL‐21	↑γH2AX, PARP, 53BP1, ↓MAD2, HL60	Oxidative stress	[121]
Brusatol	THP1, MOLM13, U937, HL60	↓Nrf2, GCLC, GCLM, HMOX‐1, NQO1	Oxidative stress	[124]
IACS‐010759	KG1, THP1, MOLM13, K562, MV4‐11, OCI‐AML3, U937, Kasumi and HL60	↓HIF‐1α	OXPHOS inhibition	[120]
Rohinitib	MOLM‐13, MOLM‐14, MV4; 11, OCI‐AML3, THP1, HL60, KASUMI‐1 and NB4	↓eIF4A, HSF1	Oxidative stress Drug resistance	[113]

Venetoclax combined with a hypomethylating agent (HMA) has emerged as an effective regimen in the treatment of early and r/r AML. HMA can generate and accumulate ROS, while activating the Nrf2 antioxidant response pathway.^50^ That leads in turn to the production of antioxidant enzymes that neutralize ROS.^97^ Nguyen et al. demonstrated that Nrf2 could be inhibited by Venetoclax, thus preventing ROS from being neutralized, as an additional mechanism of AML activity inhibition by the combination.^50^ Combined treatment with HMAs and Venetoclax enhanced mitochondrial ROS induction and apoptosis in leukemic cells compared with HMAs alone. On the other side of the analysis, Venetoclax increases the production of ROS by inhibiting the formation of respiratory chain supercomplexes, thus enhancing T cell effector functions.^98^ Azacitidine induces a virus‐mimetic response in AML cells by activating the STING/cGAS pathway, thereby making AML cells more susceptible to T cell‐mediated cytotoxicity.^99^ Lee et al. studied CD3+ CD4‐CD8‐ double‐negative T cells (DNT) as a rare subset of mature T cells. DNTs are used as an alternative to leukemia‐specific T cells, and in vivo expansion of DNTs can selectively target AML without allogeneic reactivity and non‐tumor toxicity.^100^ Venetoclax effectively enhanced the ROS function of DNT and CD8+ T cells by increasing the production of ROS. Considering NKG2D and DNAM‐1 are involved in the function of various effector immune cell subsets, the findings suggest that venetoclax may also enhance the antileukemic activity of other T‐cell or natural killer cell therapies.^101^ OXPHOS Although venetoclax combined with HMA can inhibit LSC value‐added by regulating ROS to reach the toxicity threshold and activating immune cell activity. Therefore, in r/r AML patients, venetoclax combined with azacitidine did not clear LSC. In r/r AML patients, venetoclax and azacitidine fail to eradicate LSC. Because nicotinamide metabolism is elevated in relapsed LSCs, this activates amino acid metabolism and fatty acid oxidation to drive OXPHOS, which results in diminished venetoclax and azacitidine efficacy. Jones et al. found that nicotinamide phosphoribosyltransferase is the rate‐limiting enzyme in nicotinamide metabolism that selectively eradicates r/r LSC while preserving normal HSC.^102^ Sharon et al. showed that inhibition of mitochondrial activity with protein‐synthesizing antibiotics targeting the ribosome (e.g., tedizolid) effectively overcame venetoclax resistance. Application of triple therapy with venetoclax, azacitidine, and tedizolid can effectively prevent AML resistance to improve efficacy.^103^ In addition, the application of electron transport chain complex inhibitors, pyruvate dehydrogenase inhibitors, or mitochondrial ClpP protease agonists for the treatment of venetoclax‐resistant AML. It significantly delayed disease recurrence. This finding highlights the central role of mitochondrial adaptation in AML treatment.^104^ As well as some classic drugs targeting other mechanisms of action for AML, such as mubritinib, have been found to prevent LSC proliferation through anti‐OXPHOS.^105^ In summary, venetoclax in combination with HMA can promote OXPHOS for AML. Yet, this regimen does not seem to completely bring ROS levels to the toxicity threshold. In r/r AML, this combination regimen does not offer therapeutic advantages. And, the combination alone may lead to disease relapse. Current venetoclax‐based regimens are mainly used in elderly AML patients. The goal of treatment in elderly patients is to improve the quality of survival and prolong the survival period. Consequently, every effort should be made to avoid disease recurrence during treatment. The treatment can therefore be combined with antioxidative stress drugs. It seems to be a better treatment method.

Mitochondrial dysfunction can induce oxidative stress and ROS production, which are important features of LSC.^16^ However, mitochondrial adaptations and high OXPHOS metabolism were associated with resistance to Venetoclax.^106^ And mitochondrial dysfunction has also been associated with gene mutations in AML. NPM1 is physiologically available from ribosomal biosynthesis to control MYC or TP53 signaling.^107^ The NPM1 mutation (NPM1c) impairs mitochondrial function and also prevents the formation of PML nucleosomes (NBs), regulators of mitochondrial adaptations, and key oxidative effects.^108^, ^109^ ActD is an antibiotic with clear clinical efficacy in r/r NPM1c‐AMLs. ActD targets damaged mitochondria and activates cGAS signaling to promote ROS production and thus restore NBs. PML NBs formation drives TP53 activation and oxidation in NPM1c‐AML cells.^109^ Through the mitochondrial/ROS/PML/TP53 oxidative pathway of ActD, venetoclax and the mitochondria of ActD synergistically scavenge LSC and prolong AML survival.^108^ Poor AML prognosis due to FLT3 gene mutations is also associated with ROS. The FLT3‐ITD gene can induce ROS production.^109^ FLT3‐ITD‐mutated starts a cycle of genomic instability via signal transducer and activator of transcription 5 (STAT5)/RAC1/NADPH, and the resulting increased ROS levels lead to increased DSBs and repair errors.^31^ Sallmyr et al. showed that the FLT3 inhibitor lestaurtinib inhibits FLT3‐ITD, and thus STAT5 phosphorylation decreases RAC1 activity and binding to NOX2, and ultimately reduces ROS levels.^31^ Mitochondrial OXPHOS state hyperactivity is a critical marker of AML drug resistance.^110^, ^111^ HO‐1 is one of the mitochondrial carriers. Kannan et al. studied that HO‐1 was elevated in cells carrying FLT3‐ITD compared with the FLT3 wild type. In AML cell line models, knockdown or inhibition of HO‐1 enhanced sensitivity to quizartinib. Nrf2 inhibitor (brusatol) leads to reduced HO‐1 expression and enhances the anti‐AML effect of TKI.^109^ Germon et al. showed that inhibition of NOX2 increased apoptosis in FLT3 mutant AML cells in response to FLT3 inhibitors. NOX2 inhibitors reduced phosphorylation and cysteine oxidation of signaling proteins that mediate growth and proliferation in FLT3+ AML cells. Suggests that inhibition of oxidative stress reduces FLT3 oncogenic signaling.^112^ The downstream factor of eukaryotic initiation factor 4A (eIF4A), heat shock factor 1 (HSF1), is a stress‐inducible transcription factor. HSF1 is able to down‐regulate the concentration of ROS toxicity in LSCs and thus promotes LSC growth and survival. Rohinitib (RHT) inhibition of eIF4A renders HSF1 inactivation and exerts a significant inhibitory effect on LSC (especially FLT3‐ITD+). In addition to antileukemic cellular activity, downregulation of HSF1 expression sensitized FLT3 mutant AML cells to FLT3 inhibitors. Thus, RHT and FLT3 inhibitors are highly synergistic in FLT3‐mutant AML cells.^113^

In addition, there are drugs to treat AML with FLT3 mutations by increasing ROS levels in the LSC. Zhao et al. showed that 4‐Hydroxyphenyl retinamide (4‐HPR) induced NF‐κB inhibition and ROS production. Thus, the 4‐HPR preferentially clears all FLT3‐ITD+ AML cells with less intervention in HSCs or other primitive cells.^114^ Wu et al. showed that penfluridol gradually enhanced the inhibition of FLT3‐WT and FLT3‐ITD mutant LSC with increasing concentration. Penfluridol treatment not only induces LSC apoptosis but also triggers autophagic responses such as light chain 3 (LC3) turnover and AVO formation by increasing intracellular ROS levels.^48^ In addition, melatonin can preferentially lead to excessive ROS production by FLT3‐ITD+ AML cells. The combination of melatonin and sorafenib showed highly synergistic therapeutic activity in model mouse cells carrying FLT3‐ITD+ AML, thereby inducing LSC apoptosis.^115^ Their study develops new therapeutic ideas for AML with FLT3 mutations under hypoxia or oxidative stress.

Furthermore, excessively high levels of ROS cause oxidative damage to the DNA and proteins of AML cells, leading to AML cell apoptosis.^116^ Therefore, AML cells must develop antioxidant defense systems to maintain tolerable levels even at high levels of ROS. MTH1 has been studied as a protein that prevents DNA damage in tumor cells at high ROS levels and could be a suitable molecular target for selectively causing DNA damage in tumor cells.^117^ The Sanjiv K study demonstrated that the MTH1 inhibitor TH1579 eliminated primary AML primitive cells (CD45 +) and leukemic stem cells (CD45 + Lin‐CD34 + CD38 −). TH1579 eliminates AML cells by causing mitotic arrest, increasing intracellular ROS levels, and enhancing oxidative DNA damage.^118^ Currently, there are also teams developing drugs that induce LSC ferroptosis by modulating ROS. APR‐246 causes a decrease in GSH levels, leading to an increase in ROS and lipid peroxides, and induces ferroptosis in AML cells for therapeutic purposes.^119^ There is currently a novel drug on the horizon, IACS‐010759. It is a clinical‐grade, highly potent, and selective small‐molecule inhibitor of complex I of the mitochondrial electron transport chain. IACS‐010759 safely targets glycolysis‐deficient tumors such as AML in vivo. It does not harm normal cells. IACS‐010759 is currently being evaluated in Phase 1 clinical trials in r/r AML and solid tumors.^120^

Research on the redox mechanism of AML resistance was hereby summarized. AML cells have been demonstrated to express high levels of intranuclear Nrf2, and its knockdown improves the drug sensitivity to Ara‐C and erythromycin, making it reasonable to speculate that Nrf2 plays an essential role in AML drug resistance.^121^ The mutant p53 can upregulate Nrf2 expression at the transcriptional level, thereby leading to antiapoptosis and drug resistance.^122^ High expression of inhibitor of apoptosis‐stimulating protein of p53 (iASPP) is a poor prognostic factor in AML.^123^ P53 and NF‐κB share similar binding sites on iASPP (Src homology 3 structural domain and anchor protein repeat), with iASPP preferring to bind to p53.^124^ Thus, when both of them are active, iASPP inhibits p53 more significantly than NF‐κBp65, and makes more contributions to the survival and drug resistance of AML cells. In addition, some drugs can synergize with anti‐OXPHOS drugs to treat AML with excellent efficacy (e.g., antiglucose transporter type 1 (GLUT1) drugs). Dual inhibition of GLUT1 and OXPHOS is a promising approach to treating AML.^125^ It is worth noting that in clinical practice, we may only consider the genetic or molecular target of the drug in combination with the chemotherapeutic agent, but rarely consider the adaptive relationship of the applied drug with ROS. Therefore, we may lead to a contradictory regulation of ROS between the two drugs. It is crucial to carefully consider the oxidative modulation of drugs when applying them clinically in the future.

## 7 CONCLUSION

Like other tumors, AML cells possess elevated levels of ROS. And, ROS can be closely associated with redox adaptation that promotes cell survival and drug resistance. However, more in‐depth studies are needed to more fully understand the redox effects of AML, including initiation, progression, and response to treatment. Numerous studies have revealed the double‐edged role of ROS in leukemia. Therefore, further studies are needed to explore its mechanism of action and clinical feasibility. At this point, we have some unresolved issues that need attention. (1) Is ALT present in AML? What is its mechanism of action? (2) The two drugs have opposite effects on ROS modulation when combined drugs are clinically applied to treat AML. How does this pairing affect the effectiveness of the treatment? (3) How do we select methods to regulate oxidative stress to precisely eliminate LSCs without harming HSCs? As research continues, the therapeutic approach to oxidative stress in AML is becoming more sophisticated. Regulation of ROS levels up to threshold and induction of apoptosis in LSCs would be promising strategies for the therapy of AML.

## AUTHOR CONTRIBUTIONS

**Chenyang Fan:** Conceptualization (equal); formal analysis (lead); investigation (lead); methodology (lead); resources (equal); validation (equal); visualization (lead); writing – original draft (lead). **Xiangdong Yang:** Formal analysis (equal); funding acquisition (equal); project administration (equal); resources (equal); supervision (equal); validation (equal). **Lixiang Yan:** Data curation (equal); investigation (equal); project administration (equal); resources (equal); validation (equal). **Zhexin Shi:** Conceptualization (equal); formal analysis (equal); funding acquisition (equal); project administration (equal); supervision (equal); validation (lead); visualization (equal); writing – review and editing (equal).

## FUNDING INFORMATION

The present study was supported by the National Natural Science Foundation of China (grant number 8177140217) and the Natural Science Foundation of Tianjin, China (grant number 21JCQNJC01210).

## CONFLICT OF INTEREST STATEMENT

The authors declare that there is no conflict of interest regarding the publication of this article.

## Data Availability

Data sharing is not applicable to this article as no datasets were generated or analyzed during the current study.
